# Integrated Analysis of lncRNA and mRNA Transcriptomes Reveals New Regulators of Ubiquitination and the Immune Response in Silica-Induced Pulmonary Fibrosis

**DOI:** 10.1155/2019/6305065

**Published:** 2019-01-13

**Authors:** Yao Zhou, Li He, Xiao-Dan Liu, Hua Guan, Ying Li, Rui-Xue Huang, Ping-Kun Zhou

**Affiliations:** ^1^Department of Occupational and Environmental Health, Xiangya School of Public Heath, Central South University, Changsha, Hunan Province 410078, China; ^2^Department of Radiation Biology, Beijing Key Laboratory for Radiobiology, Beijing Institute of Radiation Medicine, AMMS, Beijing 100850, China; ^3^Hunan Prevention and Treatment Center for Occupational Diseases, Changsha, China; ^4^Institute for Chemical Carcinogenesis, State Key Laboratory of Respiratory, School of Public Health, Guangzhou Medical University, Guangzhou 511436, China

## Abstract

**Objectives:**

As an epigenetic player, long noncoding RNAs (LncRNAs) have been reported to participate in multiple biological processes; however, their biological functions in silica-induced pulmonary fibrosis (SIPF) occurrence and development remain incompletely understood.

**Methods:**

Five case/control pairs were used to perform integrated transcriptomes analysis of lncRNA and mRNA. Prediction of lncRNA and mRNA functions was aided by the Gene Ontology (GO) and Kyoto Encyclopedia of Genes and Genomes (KEGG) databases. Additionally, we constructed a coexpression network of lncRNAs and mRNAs to identify targets of regulation.

**Results:**

In total, 1069 differentially expressed mRNAs and 366 lncRNAs were identified with the changes more than 2 times (p<0.05), of which 351 downregulated mRNA and 31 downregulated lncRNA were <0.5 (p<0.05) and those of 718 upregulated mRNAs and 335 upregulated lncRNA were >2 (p<0.05). The levels of 10 lncRNAs were measured via qRT-PCR; the results were consistent with the microarray data. Four genes named of FEM1B, TRIM39, TRIM32, and KLHL15 were enriched significantly with ubiquitination and immune response. Cytokine-cytokine receptor interaction was the most significantly enriched KEGG pathway in both mRNAs and lncRNAs. The coexpression network revealed that a single lncRNA can interact with multiple mRNAs, and vice versa.

**Conclusions:**

lncRNA and mRNA expression were aberrant in patients with SIPF compared to controls, indicating that differentially expressed lncRNAs and mRNAs may play critical roles in SIPF development. Our study affords new insights into the molecular mechanisms of SIPF and identifies potential biomarkers and targets for SIPF diagnosis and treatment.

## 1. Introduction

Evidence has been presented that silica dust acute and chronic exposures will trigger an inflammatory cascade, such as macrophages and alveolar epithelial cells proliferated successively, followed by inflammatory cascade; the fibrotic development will be ignited, which eventually will proceed to determine the silicosis [1, 2]. Silicosis is an irreversible and incurable pulmonary disorder; even when patients are no longer exposed to silica, the fibrosis remains progressive [3, 4]. Silica dust is widely distributed in workplaces in which drilling, grinding, and hammering activities occur [5, 6]. China has the highest silicosis burden worldwide, with more than 600,000 cases recorded over the past 30 years. Furthermore, the number of new cases is increasing, with over 24,000 deaths annually [7]. In South African gold mines, the incidence of silicosis is 13–25% in long-term miners [8]. Silicosis is also prevalent in developing countries. The CDC states that approximately 2 million US workers are currently exposed to silica; from 2001 to 2010, 1,437 deaths were attributable, in whole or in part, to silicosis; the youngest patient was aged only 19 years [9]. In the UK, over 3.2 million persons are occupationally exposed to silica [10]. Many preventative efforts have been made, including dust control and the development of personal protective equipment; in some developed countries, the incidence has thus steadily declined over the past few decades, but new cases or outbreaks are sporadically reported [11]. In developing countries, silica-induced silicosis associated with progressive lung fibrosis still remains a major concern [6, 12]. No effective therapy is available, and the molecular details of fibrotic progression remain unclear [13]. Early biomarkers are urgently required; these would aid in implementation of preventative measures and allow for early diagnosis, intervention, and treatment.

Some biomarkers aiding in early diagnosis have been identified and can be divided into exposure, effect, and susceptibility biomarkers. Serum KL-6, MMP-2, and SP-D are potential biomarkers of silicosis, with levels being significantly higher in cases than controls (p<0.05) [14]. HO-1, the enzyme catabolizing heme to bilirubin, is a recognized biomarker [15]. Silica increases the ceruloplasmin level, which thus serves as a diagnostic biomarker [16]. The serum CC16 level indicates the risk of silica exposure toxicity [17]. However, current biomarkers studies regarding lung fibrosis induced by silica mostly focus on the genes level, seldom associated with transcriptome changes, which is an emerging concern which have been reported to be potential biomarkers in the development of lung fibrosis. Recently, epigenetic research has shown that the changes in microRNA (miRNA)/long noncoding RNA (lncRNA) expression may affect the stability and translation of genes involved in silica-induced lung fibrosis. MiRNAs are short noncoding RNAs that bind to targeting mRNAs in a sequence-specific manner [18]. For example, miR-489 was increased significantly in an animal model of lung fibrosis, suppressing TGF-*β*1 synthesis (and where TGF-*β*1 is a transcription factor precipitating the inflammation associated with lung fibrosis [19]). MiR-449a inhibits fibrosis by targeting Bcl2, in turn regulating autophagy [20]. miR-19a is downregulated in the early stages of fibrosis and was proposed as a biomarker for early diagnosis [21]. The miR-146a level in bronchoalveolar lavage fluid aids in early diagnosis [22]. lncRNA research only commenced in the last decade; lncRNAs are long noncoding RNAs [23] modulating a series of important biological processes, e.g., metabolism and apoptosis, and serve also as useful biomarkers of various diseases, including silica-induced lung fibrosis. Sun et al. showed that the lncRNAs suc.77 and 2700086A05Rik regulated the progression of lung fibrosis [24]. lncRNA-ATB, first identified in 2014, was activated by TGF-*β* and induced lung fibrosis [25]. Thus, the potential utility of lncRNAs as fibrosis biomarkers requires attention, especially the investigation on human cases of silica associated fibrosis. However, such work in the context of silica-induced lung fibrosis is limited. Here, we collected peripheral blood samples from patients with silica-induced lung fibrosis followed by transcriptome analysis using a microarray; we then employed bioinformatics tools to identify changes in lncRNA levels in patients with progressive lung fibrosis; we identified the top 10 most-affected lncRNAs and further analyzed the interaction networks of lncRNAs and mRNAs. Our study may improve our mechanistic understanding of the silicosis and identifies novel diagnostic and therapeutic biomarkers.

## 2. Methods and Materials

### 2.1. Participants

We enrolled 10 subjects: 5 with phase I lung fibrosis (cases) and 5 healthy subjects who worked in the same industry but were not exposed to silica (controls). The inclusion criteria were (1) an occupational history of silica exposure and a fibrosis diagnosis based on the Chinese Pneumoconiosis Diagnosis Standards (GBZ70-2013), as agreed by at least three occupational physicians; (2) lack of complications such as tuberculosis, lung cancer, or an infection; and (3) voluntary agreement to participate. The mean age of the cases was 55±6.2 years and that of controls was 51±7.9 years; all participants were male. The silica dust concentration in the workplace was 6.43±0.77 mg/m^3^, exceeding the exposure limit of 0.2–1 mg/m^3^ (GBZ2-2002). At least 5 mL of peripheral blood was collected from each subject into a PAX gene Blood RNA tube (BD Biosciences, San Jose, CA, USA), held at room temperature (22–25°C) for at least 2 h to completely lyse all cells, placed at –70°C and immediately delivered to the Beijing Kangpusen Biotech Company for microarray analysis. The study was performed in accordance with all relevant tenets of the Declaration of Helsinki and was approved by the Ethics Committee of Xiangya School of Public Health (Approval no. XYGW-2018-11). All participants provided written informed consent.

### 2.2. Total RNA Isolation

Total RNA was isolated using the mirVanaRNA Isolation Kit following the manufacturer's instructions. Briefly, a 1.25 volume of absolute ethanol was added to each blood sample followed by mixing; the mixture was passed through a filter and the filter was washed three times with 700 *μ*L of miRNA wash solution and 500 *μ*L general wash solution; then, RNA was eluted into 100 *μ*L of elution solution at 95°C and stored at –70°C until analyzed. RNA purity and concentration were assessed using a QIAGEN RNeasy Mini Kit and by deriving a spectrophotometric ratio with the aid of a NanoDrop-2000 instrument, respectively (Thermo-Scientific, Waltham, MA, USA).

### 2.3. Microarray Analysis

In brief, microarray analysis was performed as follows: RNA/-poly-A-RNA-control mixtures were prepared by mixing test RNA samples (50–500 ng), a diluted poly-A-RNA control, and nuclease-free water; 4 *μ*L of first-strand buffer mix and 1 *μ*L of first-strand enzyme solution were then mixed and added, and first-strand was cDNA synthesized; this was followed by second-strand synthesis. Purified cDNA and second-cycle single-strand cDNA were subjected to WT cartridge hybridization, washed, and scanned using a DNA Microarray Scanner (Agilent, Santa Clara, CA, USA). Expression Console software (ver. 1.4.1) was used to adjust the raw data background and standardize microarray data.

### 2.4. Differential mRNA and lncRNA Expression and Cluster Analysis

Transcriptome Analysis Console (version 3.1, Affymetrix, Santa Clara, CA, USA) software was employed to explore the differential expression of mRNAs and lncRNAs between cases and controls; we selected RNAs exhibiting the greatest fold differences with reference to the p values. Cluster analysis [26] was used to identify genes exhibiting similar biological functions.

### 2.5. Gene Ontology and Pathway Enrichment Analysis

Gene Ontology (GO) and pathway enrichment analysis were performed to predict biological processes, cellular components, and molecular functions affected in disease. We used a controlled gene vocabulary (http://www.geneontology.org). The Kyoto Encyclopedia of Genes and Genomes (KEGG) database was employed for pathway enrichment analysis.

### 2.6. lncRNA-mRNA Coexpression Network

To explore interactions among differentially expressed lncRNAs and mRNAs in cases and controls, we prepared coexpression networks using only differentially expressed mRNAs with Pearson correlation coefficients ≥0.95 (p<0.01) and generated visual data. We included five essential lncRNAs and all essential mRNAs in a network assessment. Only differentially expressed mRNAs with Pearson correlation coefficients ≥ 0.90 (p<0.01) were used to construct the lncRNA-mRNA network and generate visual representations.

### 2.7. Quantitative Real-Time PCR (RT-PCR) Validation

Total RNA was isolated and reverse-transcribed into cDNA using a PEXBIO RNA kit according to the manufacturer's protocol. qPCR was performed with the aid of Super Real PreMix Plus (SYBR Green, Tiangen, Beijing, China) on a CFX96TM Real-Time System (Bio-Rad, Hercules, CA, USA). GAPDH served as the internal control and fold change calculations were made using the 2–^Δ  Δ  CT^ method. The primer sequences are shown in Table S1. Differences between mRNA and lncRNA expression levels were compared using Student's two-tailed t-test. A p value <0.01 was considered to reflect significance after false discovery rate correction for multiple testing.

### 2.8. Statistical Analysis

R software (R Development Core Team, Vienna, Austria) was used to compare the expression levels of mRNAs and lncRNAs, for cluster analysis, and to draw volcano maps. One-way ANOVA was employed to explore the significance of differences in mRNA and lncRNA levels after log_2_ transformation. The fold changes between cases and controls were calculated; changes >2 or <0.5 were considered to indicate differential expression; p<0.05 was taken to indicate significance.

## 3. Results

### 3.1. Comprehensive Transcriptome Analysis Reveals Differentially Expressed mRNAs and lncRNAs

Through microarray analysis, 1069 differentially expressed mRNAs were identified with the changes more than 2 times (*p*<0.05), of which one-way ANOVA showed that the fold changes of 351 downregulated mRNA were < 0.5 (*p*<0.05) and those of 718 upregulated mRNAs were > 2 (*p*<0.05). The most prominently upregulated mRNA was TC0700007850.hg.1 (fold change, 8.00;* p*<0.05). The top 25 up- and downregulated mRNAs are listed in Table 1. Meanwhile, 366 differentially expressed lncRNAs were identified with the changes more than 2 times (*p*<0.05), of which one-way ANOVA showed that the fold changes of 31 downregulated lncRNAs were <0.5 (*p*<0.05) and those of 335 upregulated lncRNAs were >2 (*p*<0.05). The most prominently downregulated lncRNA was TC1300009322.hg.1, with a fold change of 0.395 (*p*<0.05). The most prominently upregulated lncRNA was TC1700010761.hg.1, with a fold change of 6.70 (*p*<0.05). The top 25 up- and downregulated lncRNAs are listed in Table 2.

**Figure 1:**
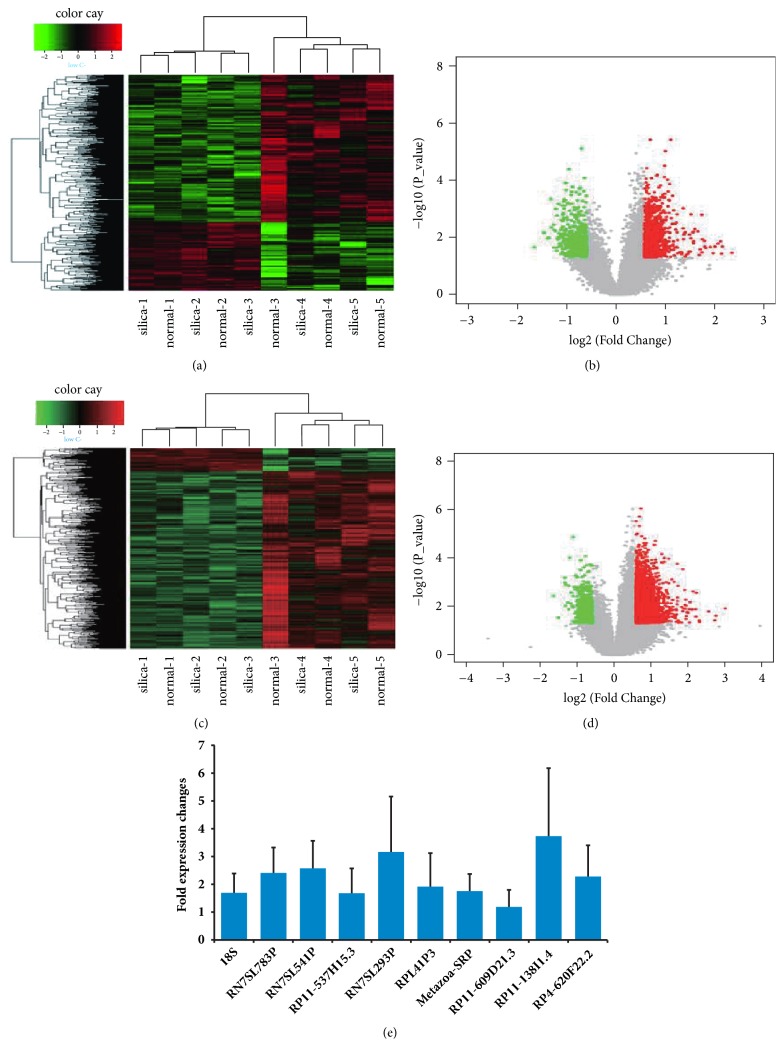
Differentially expressed mRNAs and lncRNAs among 10 samples. (a) Unsupervised cluster analysis of included differentially expressed mRNAs. Red rectangles mean the clustered upregulated mRNAs, while green rectangles mean clustered downregulated mRNAs. (b) A volcano plot of these mRNAs based on p values and fold changes. Red dots mean the upregulated mRNAs, while green dots mean downregulated mRNAs. (c) Unsupervised cluster analysis of included differentially expressed lncRNAs. Red rectangles mean the clustered upregulated lncRNAs, while green rectangles mean clustered downregulated lncRNAs. (d) A volcano plot of these mRNAs based on p values and fold changes. Red dots mean the upregulated lncRNAs, while green dots mean downregulated lncRNAs. (e) Differential expression of lncRNAs was quantified using quantitative RT-PCR.

Unsupervised cluster analysis of the mRNAs of the 10 samples, in terms of differentially expressed genes, yielded the results shown in Figure 1(a). A volcano plot of these mRNAs based on p values and fold changes is shown in Figure 1(b). Figure 1(b) shows that more mRNAs were up- than downregulated. Unsupervised cluster analysis of the lncRNAs of the 10 samples is shown in Figure 1(c). Highly expressed lncRNAs are shown in red and those expressed at low levels are in green. A volcano plot based on p values and fold changes is shown in Figure 1(d). Analysis of the normalized mRNA expression levels revealed significantly different mRNAs patterns in case group and control group (718 up- and 351 downregulated) along with more mRNAs were up- than downregulated (Figures 1(a) and 1(b)). The upregulated mRNAs with the greatest fold change over 5, which are in descending rank, were* LOYBOY, DEEZOY, SNEYSPORBY, NYSPSWBY, ZORSKABU, TOYPYBU,* and* WUSLAWBUTO*. Meanwhile, the downregulated mRNAs with the greatest fold change <0.35, which are in descending rank, were CPA3, FCER1A, and CCR5. Analysis of the normalized lncRNA expression levels revealed significantly different lncRNAs patterns in case group and control group (335 up- and 31 downregulated) along with more lncRNAs were up- than downregulated (Figures 1(c) and 1(d)). The upregulated lncRNAs with the greatest fold change over 4, which are in descending rank, were RP11-138I1.4, RP4-620F22.2, and RPL41P3. Meanwhile, the downregulated lncRNAs with the greatest fold change <0.45, which are in descending rank, were RP11-13A1.3, LOC286437, RP4-539M6.21, RP11-333J10.2, and FTX. As expected, the mRNAs and lncRNAs expression levels in Figures 1(a)–1(d) were observed to be consistent with case group and control group.

### 3.2. Comprehensive Transcriptome Functional Analysis of Differentially Expressed mRNAs and lncRNAs Reveals New Regulators of Ubiquitination and the Immune Response

**Figure 2:**
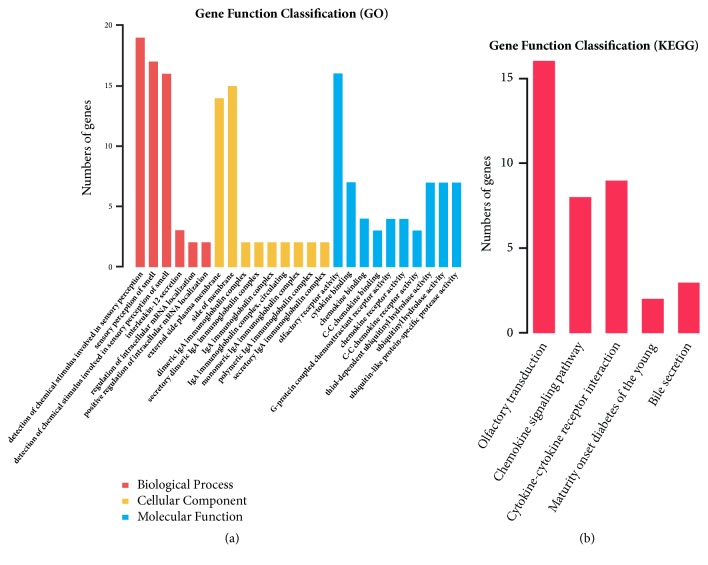
GO analysis and KEGG pathway assessment on mRNAs. (a) GO analysis on mRNAs. (b) KEGG pathway analysis on mRNAs.

**Figure 3:**
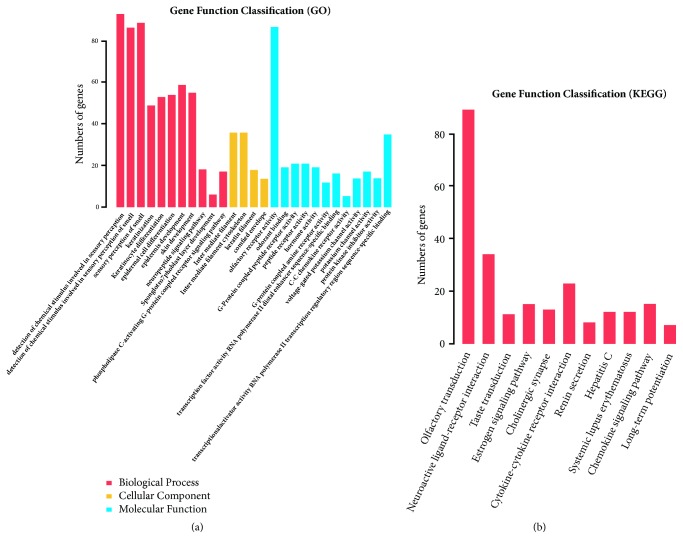
GO analysis and KEGG pathway assessment on lncRNAs. (a) GO analysis on lncRNAs. (b) KEGG pathway analysis on lncRNAs.

To highlight the biological functions and signaling pathways change in differentially expressed mRNAs and lncRNAs, we performed GO analysis and KEGG pathway assessment on mRNAs and lncRNAs, respectively (Figure 2). For mRNAs, in the main GO biological process affected was sensory perception of smell and interleukin-12 secretion, while in the affected GO cellular components were principally the immune response including secretory dimeric IgA immunoglobulin complex and monomeric IgA immunoglobulin complex, while the GO molecular functions affected were olfactory receptor activity, cytokine binding, and, in particular, thiol-dependent ubiquitinyl hydrolase activity, ubiquitinyl hydrolase activity, and ubiquitin-like protein-specific protease activity (Figure 2(a)). Four genes named FEM1B, TRIM39, TRIM32, and KLHL15 were enriched significantly with ubiquitination and immune response. KEGG pathway analysis emphasized olfactory transduction, chemokine signaling, cytokine-cytokine receptor interaction, maturity-onset diabetes of the young, and bile secretion (Figure 2(b)). GO and KEGG analyses of differentially expressed lncRNAs are shown in Figure 3. Differentially expressed lncRNAs were predicted to have the following main functions: keratinization, keratinocyte differentiation, and epidermal cell differentiation in the biological process category; intermediate filament and the GO molecular components in the cellular component category; and G-protein-coupled amine peptide receptor activity, hormonal activity, transcription factor activity, and RNA polymerase/distal enhancer sequence-specific binding in the molecular function category (Figure 3(a)). KEGG pathway analysis of all differentially expressed lncRNAs significant at p<0.05 revealed that the affected pathways included olfactory transduction, neuroactive ligand-receptor interaction, estrogen signaling, the cholinergic synapse, renin secretion, and cytokine-cytokine receptor interaction (Figure 3(b)). In order to explore individual lncRNA function in the development of lung fibrosis, we subjected the following 10 lncRNAs to individual GO and KEGG analysis: 18S_F, RPL41P3, RN7SL541P, RP11.537H15.3, RN7SL293P, RP4-620F22.2, Metazoa-SRP, RP11-609D21.3, RP11-138I1.4, and RN7SL783P. GO analysis revealed most of these selected individual lncRNAs of involvement in chemokine binding, CoA-transferase activity, and G-protein-coupled serotonin receptor activity, all of which are associated with SIPF development (Figure S1-S5); intriguingly, some lncRNAs were predicted to have immune response such as RP11.537H15.3 affected immunoglobulin production and adaptive immune response. The KEGG data showed that these selected individual lncRNAs participated in multiple pathways, including the herpes simplex infection pathway, a hepatitis C pathway involving inflammation, and the PPAR signaling pathway.

### 3.3. Verification of Expression Changes in lncRNAs

To verify the differential expression of lncRNAs, we quantified the expression levels of 10 lncRNAs in 20 cases and 20 controls, 18S_F, RPL41P3, RN7SL541P, RP11.537H15.3, RN7SL293P, RP4-620F22.2, Metazoa-SRP, RP11-609D21.3, RP11-138I1.4, and RN7SL783P, via quantitative RT-PCR. The expression levels of cases and controls differed significantly (Figure 1(e)), consistent with the microarray data.

### 3.4. LncRNA-mRNA Network Analysis

To analyze the lncRNA-mRNA coexpression interaction network, we sought genes that were significantly enriched in both cases and controls. A total of 2,053 such genes were identified, and lncRNA-mRNA coexpression networks were constructed; this identified five lncRNAs (RPL41P3, RP11-609D21.3, RP4-620F22.2, AP001610.5, and RN7SL783P) that determined the essential differences between cases and controls (Figures S6). These lncRNAs and their coexpressed mRNAs, may indicate how silica exerts biological effects and may also serve as useful therapeutic targets. For example, AP001610.5 was coexpressed with the following mRNAs/genes:* nuraro, IFIT3, TAGLN3, bawma, rasuru, IFIT1, CT45A9, nymawbo, *and* CT45A8*. RN7SL783P was coexpressed with many more mRNAs/genes, including pulmonary fibrosis-associated genes such as POYNEY and PODUBU. RP4-620F22.2, RPL41P3, and RP11-605F22.1 were also associated with many mRNAs (Table S2).

## 4. Discussion

Microarray and bioinformatics techniques now allow us to detect changes in lncRNA expression; most work to date has focused on miRNAs [27–33]. lncRNAs are longer sequence and have more complex functions than miRNAs [34]. For the first time, we herein identified mRNAs and lncRNAs that were abnormally expressed in human lung fibrosis induced by silica exposure, which exhibits complex pathological changes; no effective therapy is available. Biomarkers discovered via mRNAs and lncRNAs analysis might facilitate diagnosis and therapy [35, 36].

Differentially expressed mRNAs and lncRNAs have been reported to be associated with development of lung fibrosis. Laurent et al. explored the cultured lung fibroblast transcriptome of idiopathic pulmonary fibrosis; mRNAs encoding* LIMS2, ASB1,* and* HHAT* were upregulated and those encoding* TRANK1, IFIT1, *and* SLC15A3* were downregulated [37]. Biswas et al. found that, in cystic fibrosis,* CTFR* expression was regulated at the mRNA level [38]. Certain mRNAs associated with lung inflammation (including that encoding IL-8) were appeared to be a regulator of other miRNAs biogenesis in lung epithelial cells, showing they were potential candidates for anti-inflammatory therapeutics for cystic fibrosis [39, 40]. We found that mRNAs encoding* LOYBOY, DEEZOY, *and* SNEYSPORBY* were upregulated, and those encoding* CPA3*,* FCER1A,* and* CCR5* were downregulated; these mRNAs may be involved in silica-induced lung fibrosis. Although lncRNAs may be potential therapeutic targets in fibrosis [41], the only relevant report is that of Sun et al.; in the paraquat-induced mouse lung fibrosis model, 513 lncRNAs were upregulated and 204 were downregulated, which affected cell differentiation, epithelial morphogenesis, and immune response, all of which are closely associated with the epidermal-mesenchymal transition (EMT) [24]. Our study identified 366 differentially expressed lncRNAs in human sample in which the numbers were lower than Sun et al. study. This might be due to the species variation.

Go and KEGG analyses are commonly used to predict biological functions and/or signaling pathways affected by mRNAs and lncRNAs. On GO analysis of significant changes in mRNAs (fold change >2; p<0.05), in terms of biological proses, cellular components, and molecular function, the most-affected processes were interleukin-12 secretion, regulation of intracellular mRNA localization, effects on the dimeric IgA immunoglobulin complex, C-C chemokine binding, inflammation response, immune response, ubiquitin-like protein-specific protease activity, and thiol-dependent ubiquitinyl hydrolase activity. Here, the results which the inflammation response affected by mRNAs are consistent with the previous report; however, mRNAs affecting the ubiquitination and immune response are new insights into the mRNAs function in the lung fibrosis. This suggests mRNAs might participate in lung fibrosis process by regulating immune response and ubiquitination. Ubiquitin is a highly conserved low-molecular-weight protein and ubiquitination represents a very common posttranslational modification. Extracellular ubiquitin regulates the immune response and exhibits anti-inflammatory and neuroprotective activities. Zhou et al. reported that silica activated macrophages and increased circRNA HECTD1 levels via ubiquitination [42]. Tsubouchi et al. suggested that, in a bleomycin-induced lung fibrosis mouse model, azithromycin (AZM; a second-generation antibacterial macrolide) inhibited autophagy via ubiquitination of NOX4 [43]. Nan et al. found that ubiquitination of the transcription factors Smad2 and Smad3 was changed via the action of ubiquitin carboxyl-terminal hydrolase-L5 during the pathogenesis of idiopathic pulmonary fibrosis [44]. However, the role of ubiquitination in the development of SIPF remains poorly understood and further studies are thus required. GO analysis indicated that both mRNAs and lncRNAs participate in the immune response in lung fibrosis. Balloy V. et al. assessed transcriptomes of lncRNA between cystic fibrosis and found a specific cystic fibrosis signature of lncRNA expression was associated with immune response of patients [45]. A study by Sookoian S. et al. found that lncRNA MALAT1 participated in the development of liver fibrosis [46]. However, studies regarding the underlying mechanism of lncRNAs participating in the development of silica-induced lung fibrosis remain further investigated.

Liu et al. created an lncRNA/mRNA coexpression network for human pulmonary epithelial cells; 24 mRNAs were coexpressed and interacted with 33 lncRNAs; each mRNA interacted with a few lncRNAs and each lncRNA with a few mRNAs [47]. We included RPL41P3, RP11-609D21.39, RP4-620F22.1, AP001610.5, and RN7SL783P in a coexpression network with related mRNAs. For RPL41P3, GO analysis revealed involvement in chemokine binding, CoA-transferase activity, and G-protein-coupled serotonin receptor activity, all of which are associated with SIPF development (Figure S1); lncRNA-RP11-609D21.3 was principally associated with carbohydrate transporter, endoribonuclease, and ribonuclease A activities, and with spliceosome activity, ferroptosis, and Kaposi's sarcoma-associated herpesvirus infection (Figure S2). The lncRNARP4-620F22.2 was associated with sphinganine-1-phosphate metabolism, penetration of the zona pellucida, diol and sphingoid metabolism, sperm fusion to the egg plasma membrane, and viral RNA genome replication. Cell component analysis indicated that this lncRNA affected the exterior and marginal plasma membrane; the molecular functions involved were dihydrosphingosine-1-phosphate phosphatase, sphingosine-1-phosphate phosphatase, and hyaluronoglucosaminidase, as well as sequence-specific single-stranded DNA binding and hexosaminidase (Figure S3). The biological processes in which Metazoa-SRP lncRNA was involved included type I interferon signaling pathway, the cellular response to type I interferon, negative regulation of helicase activity, the viral defense response and its negative regulation, intracellular viral protein transport, intracellular symbiont protein transport, and cellular responses to interferon-*α* and exogenous dsRNA. The KEGG data showed that the lncRNA participated in multiple pathways, including the herpes simplex infection pathway, a hepatitis C pathway involving inflammation, and the PPAR signaling pathway (Figure S4). The lncRNA RN7SL783P molecular functions included melanocortin receptor binding and adenylate cyclase and phosphorus-oxygen lyase. The KEGG data showed that the lncRNA was active in melanogenesis, estrogen signaling, gastric acid secretion, GnRH signaling, circadian entrainment, aldosterone synthesis, and secretion, as well as inflammatory regulation of TRP channels, glucagon signaling, vascular smooth muscle contraction, oocyte meiosis apelin signaling, adrenergic signaling in cardiomyocytes, oxytocin signaling, cGMP-PKG signaling, calcium signaling, cAMP signaling, rap1 signaling, and phototransduction (Figure S5).

Our work provides an initial understanding of the roles played by lncRNAs in SIPF. The lncRNA levels determined via quantitative reverse transcription (qRT-PCR) were consistent with the microarray data. Our results suggest that (1) silica exposure modulates the expression of both lncRNAs and genes and (2) some silica-induced genes may be involved in ubiquitination, and some lncRNAs may affect immune cell interactions. The mechanisms by which lncRNA and mRNA coexpression mediates SIPF require further study.

However, our study had certain limitations. First, the lncRNAs measured via qRT-PCR constituted only a small proportion of those identified via microarray; some lncRNAs may have been missed such that further studies are required. Second, we recruited only five cases; studies with larger samples are required. However, we are the first to use human peripheral blood samples to identify lncRNAs and mRNAs in patients with SIPF.

## 5. Conclusions

Gene and lncRNA expression profiles were dysregulated in the peripheral blood of SIPF patients, suggesting future diagnostic and therapeutic biomarkers. LncRNA dysregulation may play an important role in disease development, given its plethora of effects on posttranscriptional mRNA regulation. However, further biology function work is required.

## Figures and Tables

**Table 1:** The top 25 upregulated and top 25 downregulated mRNAs from case group compared with control group.

**Upregulated mRNAs**				**Downregulated mRNAs**			
**Accession**	**Symbol**	**Fold**	***p *value**	**Accession**	**Symbol**	**Fold**	***p* value**
TC0X00007799.hg.1	loyboy	5.78	0.012	TC0300009122.hg.1	CPA3	0.30	0.025
TC1300008271.hg.1	deezoy	5.54	0.025	TC0100010252.hg.1	FCER1A	0.31	0.022
TC0300010812.hg.1	sneysporby	5.44	0.038	TC0300007257.hg.1	CCR5	0.33	0.002
TC0300006637.hg.1	nyspawby	5.31	0.016	TC1700011444.hg.1	Y_RNA	0.35	0.029
TC0600009711.hg.1	zorskabu	5.31	0.019	TC0500009637.hg.1	morflaw	0.35	0.003
TC0100009141.hg.1	toypybu	5.10	0.016	TC0300012816.hg.1	P2RY12	0.36	0.0005
TC0700012732.hg.1	wuslawbu	5.01	0.017	TC1700010782.hg.1	titari	0.36	0.007
TC0100008816.hg.1	IFI44	4.87	0.024	TC1200009872.hg.1	KLRB1	0.36	0.006
TC1700009890.hg.1	RP11-138I1.4	4.60	0.005	TC1500009459.hg.1	gozo	0.37	0.002
TC0100017874.hg.1	slokubo	4.59	0.034	TC0400008033.hg.1	sagee	0.38	0.0009
TC1800008840.hg.1	glorlo	4.46	0.016	TC0500012614.hg.1	slorskarby	0.38	0.0108
TC0100011221.hg.1	snawkobu	4.46	0.013	TC0600012379.hg.1	ELOVL4	0.38	0.009
TC0500012458.hg.1	doysheeby	4.42	0.012	TC0200012982.hg.1	veyklar	0.39	0.0107
TC0100008972.hg.1	RP4-620F22.2	4.37	0.021	TC0300007255.hg.1	CCR3	0.39	0.007
TC1400006879.hg.1	snovaw	4.35	0.048	TC0100016431.hg.1	TNFSF4	0.39	0.008
TC0500010789.hg.1	seystyby	4.32	0.037	TC0600013284.hg.1	nimime	0.39	0.009
TC2200007220.hg.1	RPL41P3	4.29	0.013	TC1200011466.hg.1	RP11-13A1.3	0.40	0.009
TC0900011875.hg.1	RNU6ATAC	4.26	0.012	TC0400010745.hg.1	CLOCK	0.40	0.0001
TC1100011736.hg.1	Gorbla	4.22	0.021	TC0100008797.hg.1	AK5	0.40	0.013
TC2000008618.hg.1	LOC100287	4.20	0.048	TC0X00006723.hg.1	CDKL5	0.40	0.003
TC1600008087.hg.1	RP11-2K6.1	4.14	0.037	TC0300010302.hg.1	kleyjubo	0.40	0.0004
TC1900011146.hg.1	poyjoy	4.08	0.002	TC0800008361.hg.1	vokey	0.40	0.0006
TC0600011130.hg.1	HIST1H4C	4.07	0.013	TC0X00010443.hg.1	LOC286437	0.40	0.0107
TC0X00007364.hg.1	SNORA11	3.99	0.021	TC1800006915.hg.1	HRH4	0.40	0.007
TC0500011210.hg.1	deychoby	3.92	0.013	TC0700010552.hg.1	snarcheeby	0.40	0.008

“-* *-* *-” presents that no symbols are available in the microarray databases, but they were actually presenting dysregulation in the case group.

**Table 2:** The top 25 upregulated and top 25 downregulated lncRNAs from case group compared with control group.

**Upregulated lncRNAs**				**Downregulated lncRNAs**			
**Accession**	**Symbol**	**Fold**	***p *value**	**Accession**	**Symbol**	**Fold**	***p* value**
TC1700009890.hg.1	RP11-138I1.4	4.603	0.013	TC1200011466.hg.1	RP11-13A1.3	0.400	0.0094
TC0100008972.hg.1	RP4-620F22.2	4.37	0.0024	TC0X00010443.hg.1	LOC286437	0.404	0.0012
TC2200007220.hg.1	RPL41P3	4.298	0.013	TC2200008440.hg.1	RP4-539M6.21	0.418	0.007
TC1700009599.hg.1	RP11-609D21.3	3.66	0.012	TC1700007659.hg.1	RP11-333J10.2	0.434	0.00009
TC1500009338.hg.1	RP11-605F22.1	3.26	0.000	TC0X00010064.hg.1	FTX	0.445	0.0026
TC1900006725.hg.1	CTC-518P12.6	3.18	0.04	TC0800011834.hg.1	ASAP1-IT1	0.458	0.0095
TC1000007371.hg.1	AP001610.5	3.12	0.02	TC1100006449.hg.1	RP11-326C3.10	0.471	0.041
TC1600011549.hg.1	skeyglerbu	3.07	0.02	TC1700011172.hg.1	RP11-257O5.2	0.471	0.0015
TC1200011218.hg.1	RP11-368L12.1	3.05	0.00	TC0800010416.hg.1	RP11-11C20.3	0.479	0.0020
TC0500006875.hg.1	RP11-1143G9.4	2.94	0.00	TC0200015562.hg.1	AC083900.1	0.483	0.0005
TC1700011014.hg.1	CTD-2139B152	2.76	0.01	TC0500013315.hg.1	CTD-2306M10.1	0.486	0.013
TC0700011233.hg.1	rerlee	2.74	0.01	TC0300008280.hg.1	LINC01215	0.487	0.003
TC1700011336.hg.1	rasleebu	2.70	0.01	TC1500009462.hg.1	RP11-519C12.1	0.487	0.0004
TC1600011191.hg.1	RP11-264B14.2	2.65	0.03	TC1200011369.hg.1	RP11-530C5.4	0.494	0.0006
TC0100009018.hg.1	GS1-21A4.1	2.58	0.02	TC0100007717.hg.1	RP11-415J8.7	0.49	0.0006
TC0600012967.hg.1	fawnoybu	2.57	0.00	TC1200006889.hg.1	RP11-180M15.7	0.49	0.005
TC0900011401.hg.1	RP1-93H18.6	2.55	0.00	TC0100018423.hg.1	LINC01355	0.50	0.002
TC0100017733.hg.1	werteebo	2.50	0.00	TC1200010249.hg.1	RP11-996F15.6	0.51	0.005
TC1400008210.hg.1	TSNAX	2.49	0.01	TC1600008532.hg.1	RP11-319G9.3	0.51	0.001
TC1000010277.hg.1	RP11-638I2.8	2.49	0.007	TC1100011979.hg.1	RP11-178H8.7	0.51	0.001
TC0300009362.hg.1	RN7SL398P	2.46	0.000	TC0300013876.hg.1	EPHB1	0.51	0.003
TC0X00009457.hg.1	RP11-3P17.5	2.45	0.04	TC1300007758.hg.1	RP11-10E18.7	0.51	0.0001
TC14_GL000009v2_ra ndom00006439.hg.1	RN7SL15P	2.43	0.000	TC0100017904.hg.1	CHRM3-AS2	0.519	0.005
				TC1100012004.hg.1	RP11-685N10.1		
TC1100009942.hg.1	RP11-435B5.6	2.43	0.02	TC1400008411.hg.1	RP11-73M18.6	0.21	0.005
TC1700008351.hg.1	TRIM22	2.42	0.02				

“-* *-* *-“ presents that no symbols are available in the microarray databases, but they were actually presenting dysregulation in the case group.

## Data Availability

The data used to support the findings of this study are included within the supplementary information file.

## Additional Points

*Key Messages.* What is already known about this subject? Silica exposure in the workplace is the potential risk factor for workers, which has been identified to be the cause of silicosis, leading to damage workers health, with many complications including tuberculosis. Preliminary studies suggest that early discovery and diagnosis of biomarkers would benefit from providing interventions on improving patients' life quality. What are the new findings? To the best of our knowledge, this study focusing on the uncovering of the transcriptomes signature of patients with silicosis is firstly reported. Previous similar studies were conducted using animal model or cell model rather than using peripheral blood samples. In total, 1069 differentially expressed mRNAs and 366 lncRNAs were identified. Four genes named FEM1B, TRIM39, TRIM32, and KLHL15 were enriched significantly with ubiquitination and immune response. How might it impact clinical practice in the foreseeable future? This study indicated that these lncRNAs and mRNAs may play a critical biological role in the progression and development of silica-induced silicosis and are thus candidate therapeutic targets and potential diagnosis biomarkers. Meanwhile, this transcriptomes study might provide more baseline understanding the molecular mechanisms of silicosis which could pave the way for a further functional study of underlying regulatory mechanisms.

## Supplementary Materials

Supplementary Materials Figure  S1. GO analysis and KEGG pathway assessment on lncRNA RP11-138I1.4. A. GO analysis on lncRNA RP11-138I1.4. B. KEGG pathway analysis on lncRNA RP11-138I1.4. Figure  S2. GO analysis on lncRNA RPL41p3. Figure  S3. GO analysis on 18S_F. Figure  S4. GO analysis and KEGG pathway assessment on lncRNA AP001610.5. A. GO analysis on lncRNA AP001610.5. B. KEGG pathway analysis on lncRNA AP001610.5. Figure  S5. GO analysis and KEGG pathway assessment on lncRNA RN7SL783P. A. GO analysis on lncRNA RN7SL783P. B. KEGG pathway analysis on lncRNA RN7SL783P. Figure  S6. lncRNA-mRNA coexpression network. Figure  S7. lncRNA-mRNA coexpression network of AP001610.5, RP4-620F22.2, RP11-605F22.1, and RP11-609D21.3, respectively. Table  S1. Primer sequences used in Quantitative Real-Time PCR (qRT-PCR). Table  S2. Five pathways and the corresponding affected genes in case group compared with control group.
