# Supplementary material for: Integrated Analysis of lncRNA and mRNA Transcriptomes Reveals New Regulators of Ubiquitination and the Immune Response in Silica-Induced Pulmonary Fibrosis
Source: Biomed Res Int. 2019 Jan 13;2019:6305065. doi: 10.1155/2019/6305065 (PMC6348882; doi:10.1155/2019/6305065)
Supplement: Supplementary Materials — Figure S1. GO analysis and KEGG pathway assessment on lncRNA RP11-138I1.4. A. GO analysis on lncRNA RP11-138I1.4. B. KEGG pathway analysis on lncRNA RP11-138I1.4. Figure S2. GO analysis on lncRNA RPL41p3. Figure S3. GO analysis on 18S_F. Figure S4. GO analysis and KEGG pathway assessment on lncRNA AP001610.5. A. GO analysis on lncRNA AP001610.5. B. KEGG pathway analysis on lncRNA AP001610.5. Figure S5. GO analysis and KEGG pathway assessment on lncRNA RN7SL783P. A. GO analysis on lncRNA RN7SL783P. B. KEGG pathway analysis on lncRNA RN7SL783P. Figure S6. lncRNA-mRNA coexpression network. Figure S7. lncRNA-mRNA coexpression network of AP001610.5, RP4-620F22.2, RP11-605F22.1, and RP11-609D21.3, respectively. Table S1. Primer sequences used in Quantitative Real-Time PCR (qRT-PCR). Table S2. Five pathways and the corresponding affected genes in case group compared with control group. [file 6305065.f1.zip › supplementary Materials tables.pdf]

“---“ presents no symbols are available in the microarray databases, but they were actually presenting dysreguation in the case group

Table S1 Primer sequences used in Quantitative real-time PCR(qRT-PCR)

**lncRNA**	**Primer**
GAPDH_F	GGAGCGAGATCCCTCCAAAAT
GAPDH_R	GGCTGTTGTCATACTTCTCATGG
18S_F	GCGGCGGAAAATAGCCTTTG
18S_R	GATCACACGTTCCACCTCATC
RN7SL783P_F	AGGAGACCACCAGGTTGACTAAG G
RN7SL783P_R	AGTGGCTATTCACAGGCACGATT C
RN7SL541P_F	CCTGTAGTCTCTGGAGGCTGAGG
RN7SL541P_R	ATTGCCTGCTATGTTCCGACCTG
RP11.537H15.3_F	AGTGCTGAGAACTTCCTTGCTTCC
RP11.537H15.3_R	TGCTTGAGTTGCAGTGTAGGTTGG
RN7SL293P_F	GCTACTTGGGAAGCTGAGGC
RN7SL293P_R	TCCCTCATTCCCGGAAGGTC
RPL41P3_F	CTCTGTGCCATGAGAGCCAAGTG
RPL41P3_R	GGCCTCCACGGTGCAACAAG
Metazoa-SRP_F2	GCGGACGGATCACCTGAG
Metazoa-SRP_R2	CCCAGCCATTATGCCAGACT
RP11-609D21.3_F2	ACAAGACCACCACAGCAAGT
RP11-609D21.3_R2	TACACTCCAGTCTTTGGGCA
RP11-138I1.4_F2	TAATGCCATCACTGGGCACT
RP11-138I1.4_R2	GGCAAGCAGCTGGTCCTG
RP4-620F22.2_F2	TAACAGTCCTCTGGGCGCTG
RP4-620F22.2_R2	AGAAACTTAGGCAGTGCTGGG

TableS2. 5 pathways and the corresponding affected genes in case group compared with control group

**pathways**	Gene Ratio	*P* value	***Gene Symbol***
Olfactory transduction	16/84	1.55E-05	OR5P3/OR10V1/OR1A1/OR4A47/OR2G3/OR4C12/OR2T3/OR8G5/OR10H3/OR6C3/OR52H1/OR11H1/OR1C1/OR2T12/OR5AC2/OR2A12
Chemokine signaling pathway	8/84	0.001143	ADCY2/KRAS/CCL5/CCR4/PPBP/CCR7/CX3CR1/CCR6
Cytokine-cytokine receptor interaction	9/84	0.003407	CCL5/TNFRSF9/CCR4/TNFRSF10D/FASLG/PPBP/CCR7/CX3CR1/CCR6
Maturity onset diabetes of the young	2/84	0.034816	PDX1/NKX6-1
Bile secretion	3/84	0.046672	ADCY2/AQP8/SLC10A1
