# Supplementary figures and images for: Integrated Analysis of lncRNA and mRNA Transcriptomes Reveals New Regulators of Ubiquitination and the Immune Response in Silica-Induced Pulmonary Fibrosis
Source: Biomed Res Int. 2019 Jan 13;2019:6305065. doi: 10.1155/2019/6305065 (PMC6348882; doi:10.1155/2019/6305065)

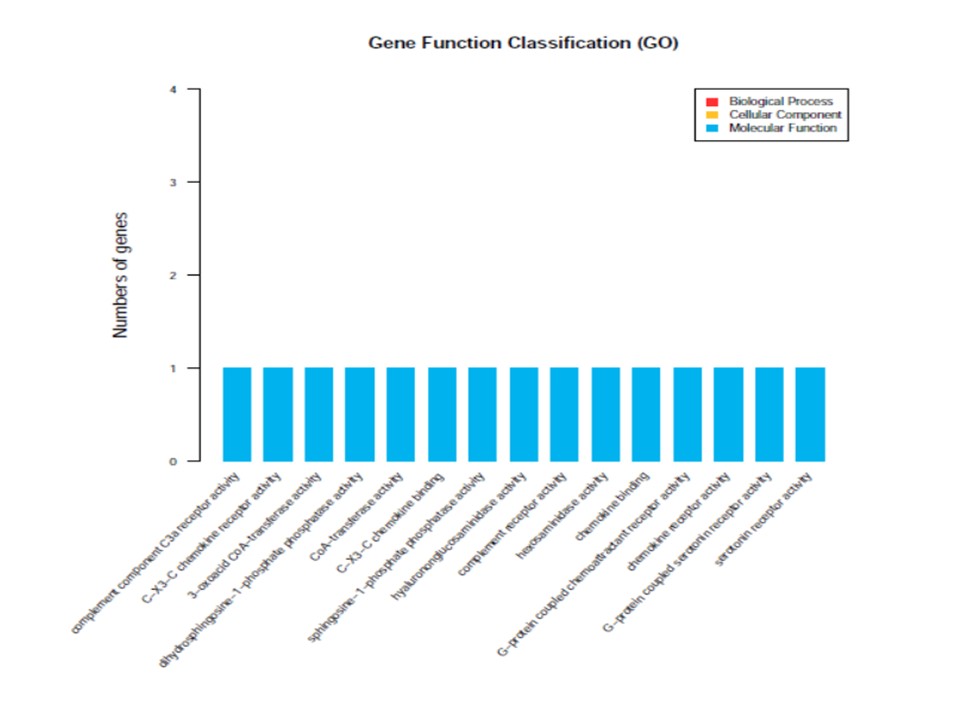

Supplement: Supplementary Materials — Figure S1. GO analysis and KEGG pathway assessment on lncRNA RP11-138I1.4. A. GO analysis on lncRNA RP11-138I1.4. B. KEGG pathway analysis on lncRNA RP11-138I1.4. Figure S2. GO analysis on lncRNA RPL41p3. Figure S3. GO analysis on 18S_F. Figure S4. GO analysis and KEGG pathway assessment on lncRNA AP001610.5. A. GO analysis on lncRNA AP001610.5. B. KEGG pathway analysis on lncRNA AP001610.5. Figure S5. GO analysis and KEGG pathway assessment on lncRNA RN7SL783P. A. GO analysis on lncRNA RN7SL783P. B. KEGG pathway analysis on lncRNA RN7SL783P. Figure S6. lncRNA-mRNA coexpression network. Figure S7. lncRNA-mRNA coexpression network of AP001610.5, RP4-620F22.2, RP11-605F22.1, and RP11-609D21.3, respectively. Table S1. Primer sequences used in Quantitative Real-Time PCR (qRT-PCR). Table S2. Five pathways and the corresponding affected genes in case group compared with control group. [file 6305065.f1.zip › figure S 2_BMRI_2622825.docx]

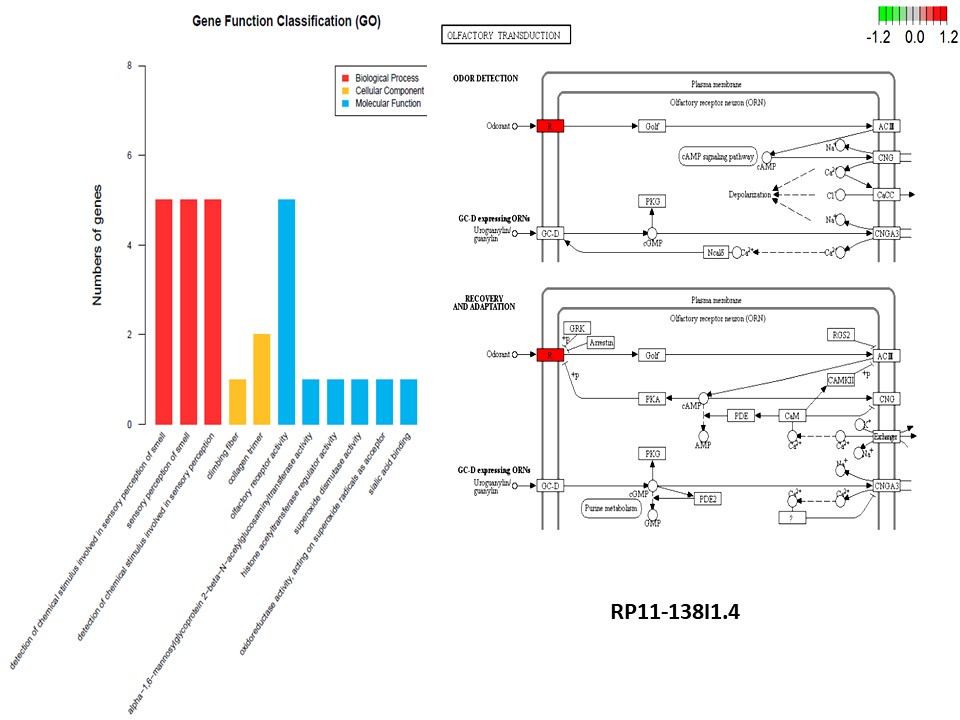

Supplement: Supplementary Materials — Figure S1. GO analysis and KEGG pathway assessment on lncRNA RP11-138I1.4. A. GO analysis on lncRNA RP11-138I1.4. B. KEGG pathway analysis on lncRNA RP11-138I1.4. Figure S2. GO analysis on lncRNA RPL41p3. Figure S3. GO analysis on 18S_F. Figure S4. GO analysis and KEGG pathway assessment on lncRNA AP001610.5. A. GO analysis on lncRNA AP001610.5. B. KEGG pathway analysis on lncRNA AP001610.5. Figure S5. GO analysis and KEGG pathway assessment on lncRNA RN7SL783P. A. GO analysis on lncRNA RN7SL783P. B. KEGG pathway analysis on lncRNA RN7SL783P. Figure S6. lncRNA-mRNA coexpression network. Figure S7. lncRNA-mRNA coexpression network of AP001610.5, RP4-620F22.2, RP11-605F22.1, and RP11-609D21.3, respectively. Table S1. Primer sequences used in Quantitative Real-Time PCR (qRT-PCR). Table S2. Five pathways and the corresponding affected genes in case group compared with control group. [file 6305065.f1.zip › figure S1_BMRI_2622824.docx]

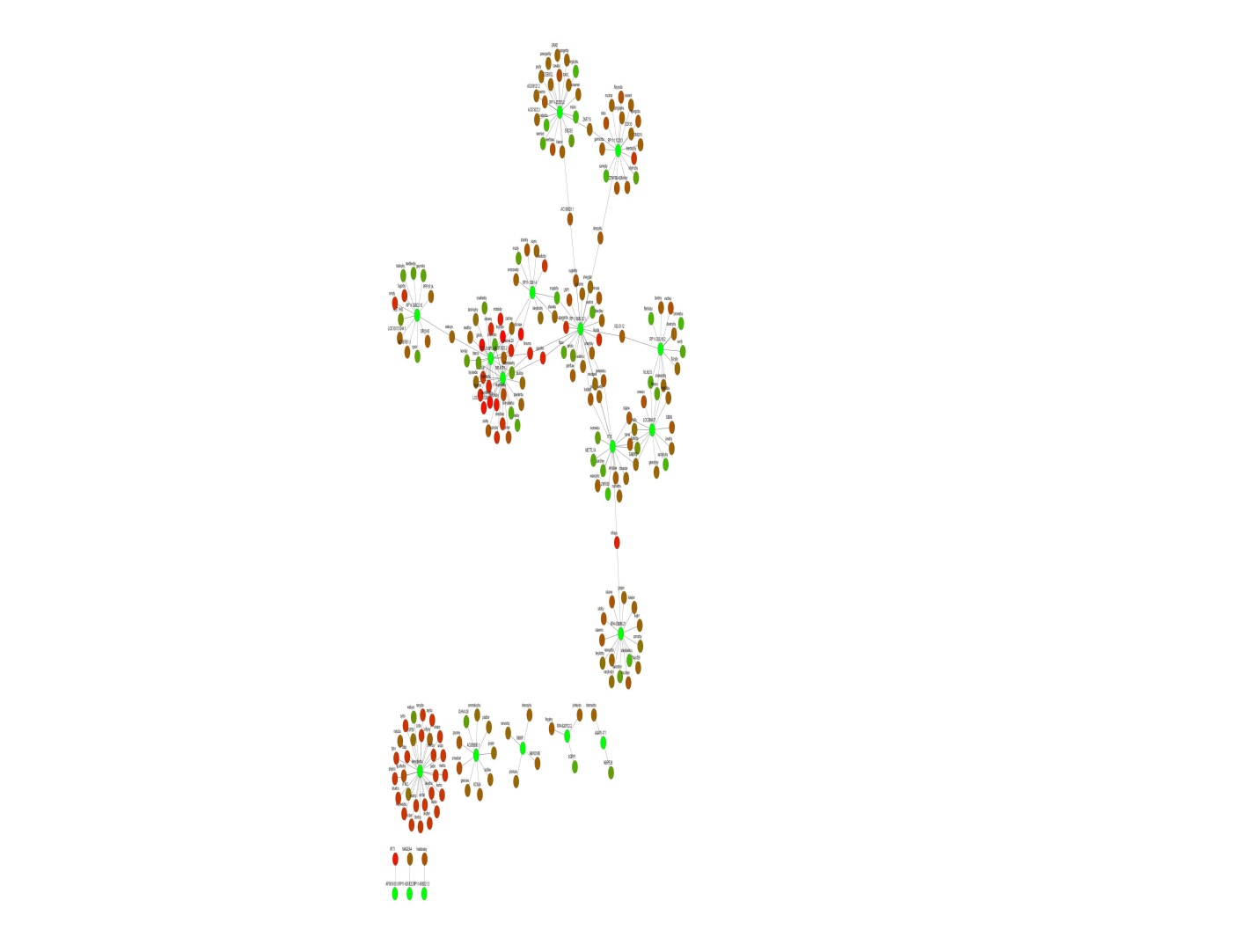

Supplement: Supplementary Materials — Figure S1. GO analysis and KEGG pathway assessment on lncRNA RP11-138I1.4. A. GO analysis on lncRNA RP11-138I1.4. B. KEGG pathway analysis on lncRNA RP11-138I1.4. Figure S2. GO analysis on lncRNA RPL41p3. Figure S3. GO analysis on 18S_F. Figure S4. GO analysis and KEGG pathway assessment on lncRNA AP001610.5. A. GO analysis on lncRNA AP001610.5. B. KEGG pathway analysis on lncRNA AP001610.5. Figure S5. GO analysis and KEGG pathway assessment on lncRNA RN7SL783P. A. GO analysis on lncRNA RN7SL783P. B. KEGG pathway analysis on lncRNA RN7SL783P. Figure S6. lncRNA-mRNA coexpression network. Figure S7. lncRNA-mRNA coexpression network of AP001610.5, RP4-620F22.2, RP11-605F22.1, and RP11-609D21.3, respectively. Table S1. Primer sequences used in Quantitative Real-Time PCR (qRT-PCR). Table S2. Five pathways and the corresponding affected genes in case group compared with control group. [file 6305065.f1.zip › figure S6_BMRI_2622835.docx]

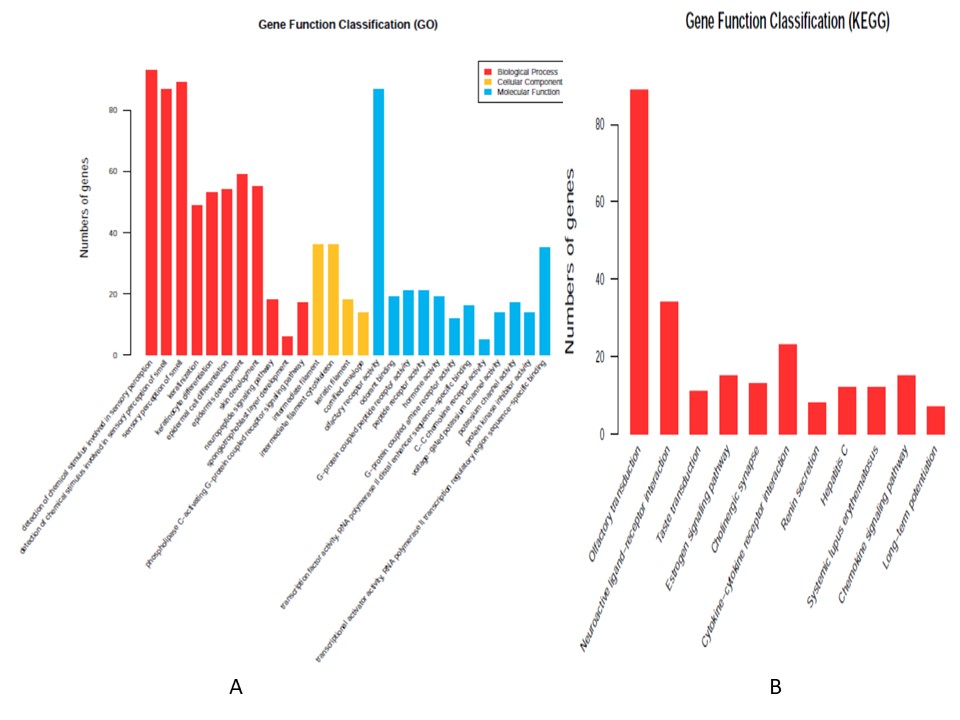

Supplement: Supplementary Materials — Figure S1. GO analysis and KEGG pathway assessment on lncRNA RP11-138I1.4. A. GO analysis on lncRNA RP11-138I1.4. B. KEGG pathway analysis on lncRNA RP11-138I1.4. Figure S2. GO analysis on lncRNA RPL41p3. Figure S3. GO analysis on 18S_F. Figure S4. GO analysis and KEGG pathway assessment on lncRNA AP001610.5. A. GO analysis on lncRNA AP001610.5. B. KEGG pathway analysis on lncRNA AP001610.5. Figure S5. GO analysis and KEGG pathway assessment on lncRNA RN7SL783P. A. GO analysis on lncRNA RN7SL783P. B. KEGG pathway analysis on lncRNA RN7SL783P. Figure S6. lncRNA-mRNA coexpression network. Figure S7. lncRNA-mRNA coexpression network of AP001610.5, RP4-620F22.2, RP11-605F22.1, and RP11-609D21.3, respectively. Table S1. Primer sequences used in Quantitative Real-Time PCR (qRT-PCR). Table S2. Five pathways and the corresponding affected genes in case group compared with control group. [file 6305065.f1.zip › figure3_BMRI_2622823.docx]

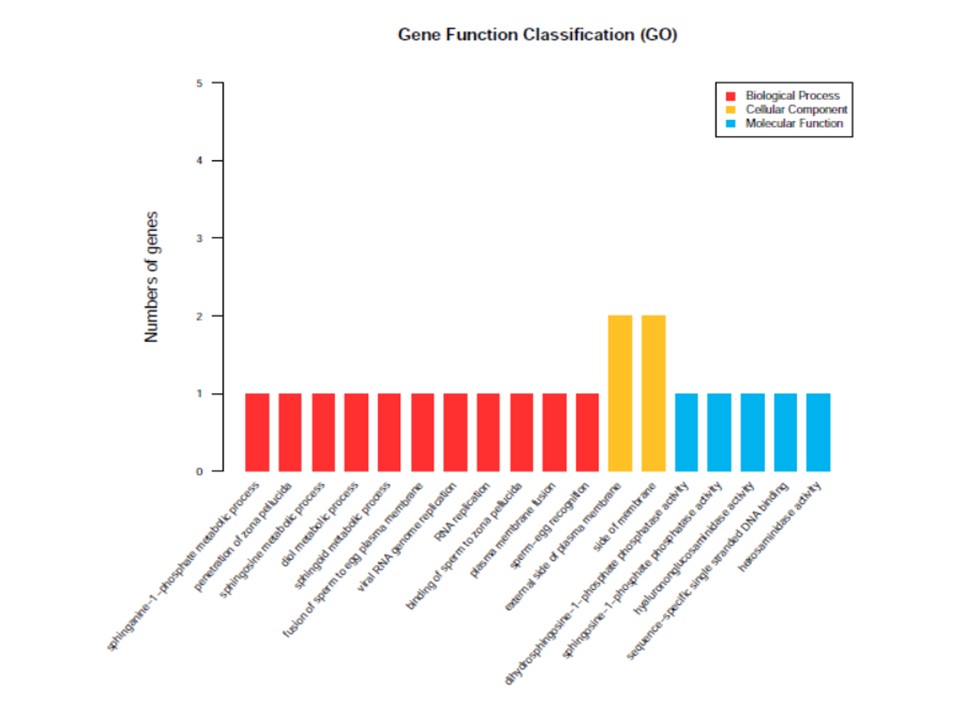

Supplement: Supplementary Materials — Figure S1. GO analysis and KEGG pathway assessment on lncRNA RP11-138I1.4. A. GO analysis on lncRNA RP11-138I1.4. B. KEGG pathway analysis on lncRNA RP11-138I1.4. Figure S2. GO analysis on lncRNA RPL41p3. Figure S3. GO analysis on 18S_F. Figure S4. GO analysis and KEGG pathway assessment on lncRNA AP001610.5. A. GO analysis on lncRNA AP001610.5. B. KEGG pathway analysis on lncRNA AP001610.5. Figure S5. GO analysis and KEGG pathway assessment on lncRNA RN7SL783P. A. GO analysis on lncRNA RN7SL783P. B. KEGG pathway analysis on lncRNA RN7SL783P. Figure S6. lncRNA-mRNA coexpression network. Figure S7. lncRNA-mRNA coexpression network of AP001610.5, RP4-620F22.2, RP11-605F22.1, and RP11-609D21.3, respectively. Table S1. Primer sequences used in Quantitative Real-Time PCR (qRT-PCR). Table S2. Five pathways and the corresponding affected genes in case group compared with control group. [file 6305065.f1.zip › figureS3_BMRI_2622826.docx]

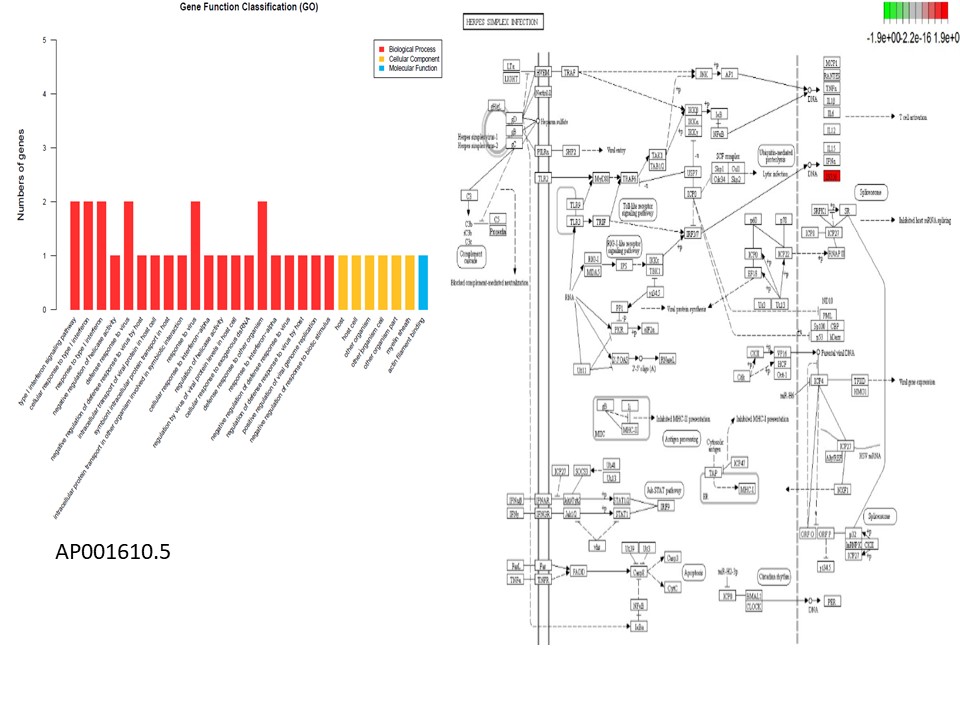

Supplement: Supplementary Materials — Figure S1. GO analysis and KEGG pathway assessment on lncRNA RP11-138I1.4. A. GO analysis on lncRNA RP11-138I1.4. B. KEGG pathway analysis on lncRNA RP11-138I1.4. Figure S2. GO analysis on lncRNA RPL41p3. Figure S3. GO analysis on 18S_F. Figure S4. GO analysis and KEGG pathway assessment on lncRNA AP001610.5. A. GO analysis on lncRNA AP001610.5. B. KEGG pathway analysis on lncRNA AP001610.5. Figure S5. GO analysis and KEGG pathway assessment on lncRNA RN7SL783P. A. GO analysis on lncRNA RN7SL783P. B. KEGG pathway analysis on lncRNA RN7SL783P. Figure S6. lncRNA-mRNA coexpression network. Figure S7. lncRNA-mRNA coexpression network of AP001610.5, RP4-620F22.2, RP11-605F22.1, and RP11-609D21.3, respectively. Table S1. Primer sequences used in Quantitative Real-Time PCR (qRT-PCR). Table S2. Five pathways and the corresponding affected genes in case group compared with control group. [file 6305065.f1.zip › figureS4_BMRI_2622827.docx]

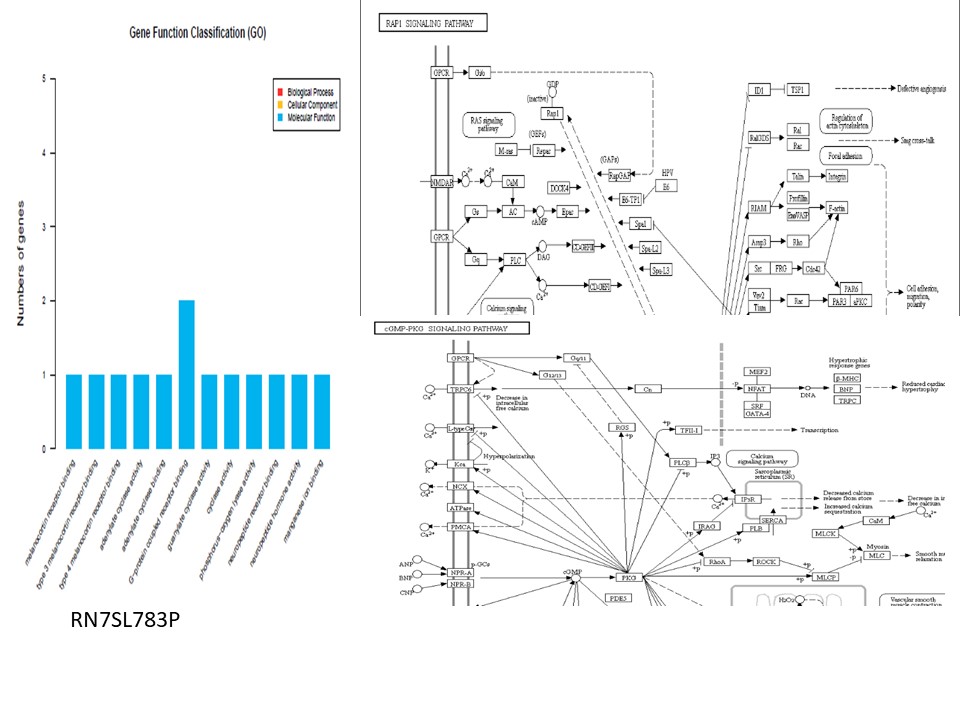

Supplement: Supplementary Materials — Figure S1. GO analysis and KEGG pathway assessment on lncRNA RP11-138I1.4. A. GO analysis on lncRNA RP11-138I1.4. B. KEGG pathway analysis on lncRNA RP11-138I1.4. Figure S2. GO analysis on lncRNA RPL41p3. Figure S3. GO analysis on 18S_F. Figure S4. GO analysis and KEGG pathway assessment on lncRNA AP001610.5. A. GO analysis on lncRNA AP001610.5. B. KEGG pathway analysis on lncRNA AP001610.5. Figure S5. GO analysis and KEGG pathway assessment on lncRNA RN7SL783P. A. GO analysis on lncRNA RN7SL783P. B. KEGG pathway analysis on lncRNA RN7SL783P. Figure S6. lncRNA-mRNA coexpression network. Figure S7. lncRNA-mRNA coexpression network of AP001610.5, RP4-620F22.2, RP11-605F22.1, and RP11-609D21.3, respectively. Table S1. Primer sequences used in Quantitative Real-Time PCR (qRT-PCR). Table S2. Five pathways and the corresponding affected genes in case group compared with control group. [file 6305065.f1.zip › figureS5_BMRI_2622828.docx]

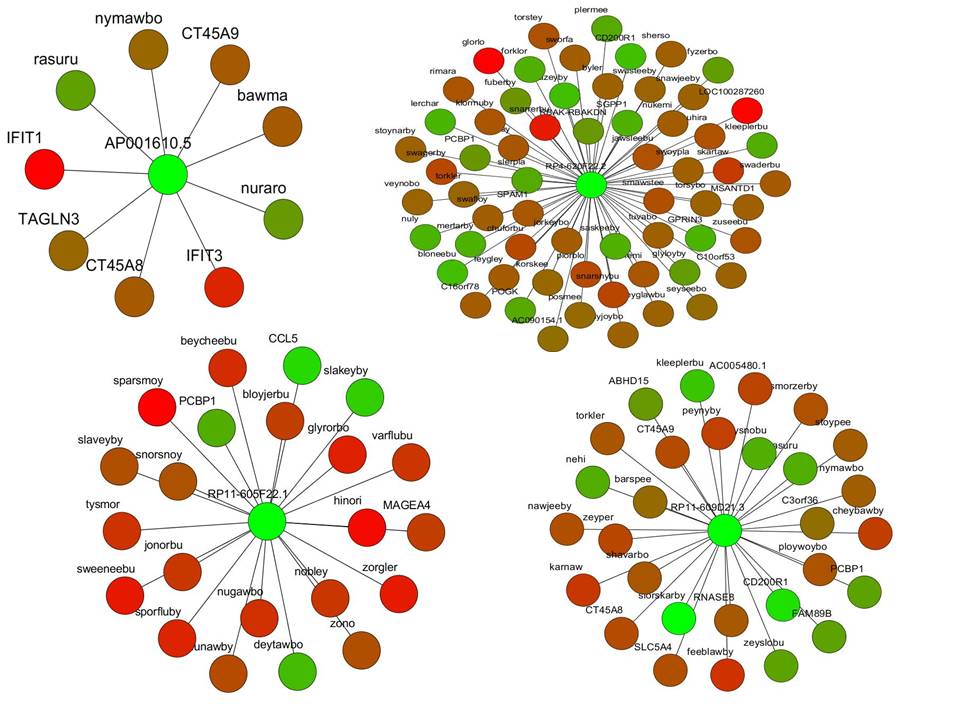

Supplement: Supplementary Materials — Figure S1. GO analysis and KEGG pathway assessment on lncRNA RP11-138I1.4. A. GO analysis on lncRNA RP11-138I1.4. B. KEGG pathway analysis on lncRNA RP11-138I1.4. Figure S2. GO analysis on lncRNA RPL41p3. Figure S3. GO analysis on 18S_F. Figure S4. GO analysis and KEGG pathway assessment on lncRNA AP001610.5. A. GO analysis on lncRNA AP001610.5. B. KEGG pathway analysis on lncRNA AP001610.5. Figure S5. GO analysis and KEGG pathway assessment on lncRNA RN7SL783P. A. GO analysis on lncRNA RN7SL783P. B. KEGG pathway analysis on lncRNA RN7SL783P. Figure S6. lncRNA-mRNA coexpression network. Figure S7. lncRNA-mRNA coexpression network of AP001610.5, RP4-620F22.2, RP11-605F22.1, and RP11-609D21.3, respectively. Table S1. Primer sequences used in Quantitative Real-Time PCR (qRT-PCR). Table S2. Five pathways and the corresponding affected genes in case group compared with control group. [file 6305065.f1.zip › figureS7_BMRI_2622836.docx]
